# Brain-derived neurotrophic factor and cytokines as predictors of cognitive impairment in adolescent and young adult cancer patients receiving chemotherapy: a longitudinal study

**DOI:** 10.1186/s12885-025-14430-3

**Published:** 2025-07-01

**Authors:** Julia Trudeau, Ding Quan Ng, Michael Sayer, Chia Jie Tan, Yu Ke, Raymond J. Chan, Alexandre Chan

**Affiliations:** 1 Department of Clinical Pharmacy Practice, University of California Irvine, 802 W Peltason Dr, Irvine, CA 92697-4625 USA; 2 Department of Pharmacotherapy, University of Utah, Salt Lake City, UT USA; 3 Division of Supportive and Palliative Care, National Cancer Centre Singapore, Singapore, Singapore; 4 Caring Futures Institute, College of Nursing and Health Sciences, Flinders University, Adelaide, Australia

**Keywords:** Cancer-related cognitive impairment, Cytokines, BDNF, Adolescent and young adult cancer patients

## Abstract

**Background:**

Inflammatory signaling is linked with cancer-related cognitive impairment (CRCI), potentially through modulation of brain-derived neurotrophic factor (BDNF) expression. Here, we evaluate associations between plasma cytokines and BDNF and their relationship with cognition in a longitudinal study of adolescent and young adult cancer patients (AYAC) receiving chemotherapy and non-cancer controls (NC) (Clinicaltrials.gov: NCT03476070).

**Methods:**

Newly diagnosed AYAC (15–39 years old) and age-matched NC completed the Functional Assessment of Cancer Therapy-Cognitive Function questionnaire (FACT‐Cog), the Cambridge Neuropsychological Test Automated Battery (CANTAB), and blood draws every 3–6 months up to 12 months (AYAC) or 6 months (NC) from baseline. Plasma levels of cytokines and BDNF were quantified using a multiplexed immunoassay and ELISA, respectively. Biomarker-cognition and cytokine-BDNF associations were analyzed using mixed-effects models with interactions for chemotherapy status for AYAC (during chemotherapy vs. > 30 days post-chemotherapy).

**Results:**

One-hundred and seventy-seven participants were included, with 66 AYAC and 111 NC. AYAC had a higher frequency of clinically significant cognitive impairment during and post-chemotherapy compared to NC. In trends unique to AYAC, higher IL-10 was associated with better self-perceived cognition, IL-8 with better multi-tasking, IL-6 with worse multi-tasking, response speed, and attention, and TNF-α with better memory (*p* < 0.05). Higher BDNF was associated with better memory and response speed (*p* < 0.05). IL-4, IL-10, TNF-α, and IFN-γ were associated with BDNF levels among AYAC and NC (*p* < 0.05).

**Conclusions:**

Our large, age-matched study implicates dysregulated cytokine signaling and altered BDNF expression in CRCI among AYAC during and post-chemotherapy. As precision medicine becomes integrated into AYA patient care, plasma BDNF and cytokines may serve as important predictors of CRCI onset.

**Trial registration:**

The study was prospectively registered on ClinicalTrials.gov (NCT03476070) on March 3, 2018.

**Supplementary Information:**

The online version contains supplementary material available at 10.1186/s12885-025-14430-3.

## Background

Cognitive toxicity experienced by cancer patients and survivors can manifest as impairments in memory, concentration, and executive function [1]. Previously referred to in the literature as “chemobrain”, growing evidence of cognitive deficits prior to therapy initiation suggests that the causes of cancer-related cognitive impairment (CRCI) are yet to be fully elucidated [2, 3].

One hypothesized mechanism of CRCI implicates the dysregulation of inflammatory cytokine signaling [3–5]. Dysregulated inflammatory signaling can be a result of cytokines released by cells in the tumor microenvironment, as well as in response to chemotherapeutic agents [3, 4]. Cytokines can cross the blood–brain-barrier (BBB) and induce tissue damage, cumulating in cognitive impairment by a variety of mechanisms such as further activation of inflammatory responses and induction of reactive oxygen species [3, 4, 6]. Studies encompassing patients with various cancer diagnoses and treatment regimens indicate associations between plasma cytokine levels and cognition [6–11], particularly the proinflammatory cytokines interleukin (IL)−6 and tumor-necrosis factor (TNF)-α [6, 9]. Preclinical evidence suggests these cytokines may mediate altered hippocampal volume and verbal memory difficulties following chemotherapy [12]. Other peripheral cytokines with potential roles in cognition such as IL-2, IL-4, IL-8, IL-10, granulocyte–macrophage colony-stimulating factor (GM-CSF), and interferon (IFN)-ƴ have been investigated as potential biomarkers of CRCI, but inconsistent results across studies have prevented a clear consensus [3, 4, 8, 13].

We hypothesize that the role of inflammatory cytokine signaling in mediating neuroinflammation is interconnected with brain-derived neurotrophic factor (BDNF), which is well-documented for its neuroprotective roles such as the regulation of synaptic plasticity [14, 15]. Circulating BDNF levels have been shown to decline over the course of chemotherapy, and lower levels were found to be associated with worse cognitive outcomes among cancer patients in several studies [14, 16, 17]. Preclinical studies suggest that dysregulated cytokine signaling can suppress BDNF expression, and this could serve as a mechanism underlying CRCI [18, 19]. While the relationship between levels of specific cytokines with BDNF in human studies remains largely inconsistent across conditions, we previously reported differential associations between cytokines and BDNF among breast cancer patients with different trajectories of CRCI [20].

Notably, both cytokine-cognition and cytokine-BDNF associations remain largely inconsistent across studies [6, 20]. Various confounders such as age-related cognitive decline, depression, and other comorbidities can make it difficult to attribute these associations to cancer or anti-cancer therapies [6, 21, 22]. Furthermore, findings from our previous studies may have limited interpretability due to a lack of a non-cancer control group for relevant comparison [1, 8, 20].

Studying cognition and biomarker levels among adolescent and young adult cancer patients (AYAC), defined as those between the ages of 15 and 39 years old, can limit confounders associated with age-related comorbidities and provide clearer insight on observed associations [23]. Understanding underlying mechanisms of CRCI in AYAC can also aid in the development of therapeutic interventions, which is of great importance for the AYAC population. Preliminary data suggests that as many AYAC are continuing their education or at critical timepoints in their careers, attention and memory deficits significantly interfere with their daily functioning [24, 25]. A recent scoping review estimated one out of four AYA cancer survivors self-report CRCI [26]. While a cross-sectional study implicated impairments in executive function and processing speed [27], longitudinal studies identifying specific cognitive domains are lacking [26]. This gap highlights the need for longitudinal studies with both self-reported and objective cognitive data to identify reliable biomarkers of CRCI among AYAC.

To our knowledge, cytokines as CRCI predictors and associations between cytokines with BDNF have not been examined longitudinally in AYAC. We previously reported baseline data from AYAC and non-cancer controls enrolled in the Adolescent and Young Adult Cancer Patients: Cognitive Toxicity on Survivorship (ACTS) study, as well as associations between BDNF with cognition from six months post-baseline [2, 14]. Here, we investigate associations of plasma cytokine and BDNF levels with longitudinal changes in objective and self-perceived cognition among AYAC during and post-chemotherapy. Associations are compared with non-cancer controls to assess their specificity to AYAC. Furthermore, we also assess associations between cytokine and BDNF levels to explore potential shared pathways.

## Methods

### Study design and participants

This is an analysis of the Adolescent and Young Adult Cancer Patients: Cognitive Toxicity on Survivorship (ACTS) study, previously described in Chan et al. [2] ACTS was a multicenter, prospective, longitudinal, observational study conducted at three hospitals that offer AYAC care between June 2018 and January 2023. The study received ethics approval from the SingHealth Centralized Institutional Review Board (CIRB 2017/3139) and informed consent was obtained prior to study participation (ClinicalTrials.gov: NCT03476070).

Two groups of participants, AYAC and non-cancer controls (NC), were recruited for this study. AYAC were between ages 15–39, newly diagnosed, treatment naïve, and capable of providing informed consent (with parental consent if < 18 years old). Participants were excluded if there was evidence of psychosis or underlying neuropsychiatric illness that could impair cognitive abilities. Recruitment occurred immediately following initial cancer diagnosis by medical oncologists, and all study procedures were conducted during appointments before patients received prescribed treatments. NC were community controls recruited through advertisement, word of mouth and patient referral. In the overall study, NC were age-matched to AYAC within three years (1:1 and 1:2 random matching ratios), with the same eligibility criteria aside from the cancer diagnosis.

AYAC were assessed at baseline prior to receiving treatment, and then every three months for up to one year post-baseline. For the purpose of this analysis, only AYAC who later received chemotherapy during the study period were included. Given the heterogeneity in treatment regimens during the 3–12 month follow-up period, post-baseline assessments for AYAC were categorized as during active chemotherapy (within 30 days of last dose) or post-chemotherapy (> 30 days after last dose) to provide clinically interpretable results. For feasibility purposes and the expectation of lower variability over time, NC were assessed at baseline and six months post-baseline. Participants without any post-baseline assessments were excluded. Assessments included tests and questionnaires administered by a trained research assistant, and relevant clinical and demographic data was collected through interviews and medical records.

### Cognitive Outcomes

#### Objective cognition

Objective cognitive function was assessed using the Cambridge Neuropsychological Test Automated Battery (CANTAB) [28, 29] administered on a tablet measuring cognitive domains of multitasking (multitasking test), memory (paired associates learning), response speed (reaction time), executive function (spatial working memory), and attention (rapid visual information processing).

To assess post-baseline objective cognition, reliable change indices (RCIs) were calculated for each cognitive domain using the change in score relative to baseline divided by the standard error of measurement of the difference based on the NC group, consistent with recommendations from the International Cognition and Cancer Task Force [1, 30, 31]. Scores were adjusted so that for all domains, a negative RCI indicates a decline from baseline.

At each follow-up assessment, participants were classified as having objective cognitive impairment if there was significant deterioration in at least one cognitive domain, defined as an RCI of < −1.96. As a pre-established statistical threshold for meaningful change in neuropsychological testing, this corresponds to a < 5% probability of deteriorating by chance [31].

#### Self-perceived cognition

The Functional Assessment of Cancer Therapy-Cognitive Function version 3 (FACT-Cog) [32–34] assesses self-perceived cognition and has previously been used in the AYAC population [24]. Thirty-seven items on a five-point Likert scale encompass four sub-scales: Perceived Cognitive Impairments (20 items), Impact of Perceived Cognitive Impairments on Quality of Life (4 items), Comments from Others (4 items), and Perceived Cognitive Abilities (9 items). Scores from the four sub-scales were summed to provide a total score (0–148) where higher scores indicate better self-perceived cognition.

To assess self-perceived cognition during follow-up, the change in total FACT-Cog score relative to baseline was calculated for each assessment. At each post-baseline assessment, participants were classified as having self-perceived cognitive impairment based on the minimal clinically important difference (MCID) of a ≥ 10.6-point decline in the FACT-Cog total score relative to baseline [35].

### Psychological distress and fatigue

As possible confounders known to impact cognition [36, 37], participants’ psychological distress and fatigue were assessed using the Rotterdam Symptom Checklist (RSCL) psychological distress subscale [38–40] and the Multidimensional Fatigue Symptom Inventory-Short Form (MFSI-SF) [41, 42], respectively.

The RSCL is designed to assess symptom burden in cancer patients [38, 39]. Items on a four-point Likert scale encompass four sub-scales: physical distress (23 items), psychological distress (7 items), activity level (8 items), and overall valuation of life (1 item). Summing the psychological distress sub-scale items provides a score (7–28), with higher scores indicating greater psychological distress.

The MFSI-SF assesses fatigue in cancer patients [41]. Thirty items on a four-point Likert scale encompass five sub-scales with six items each: general fatigue, physical fatigue, emotional fatigue, mental fatigue, and vigor. The total score is obtained by summing all the dimension except the vigor domain which is subtracted. The total score ranges from − 24 to 96, with higher scores indicating worse fatigue.

### Biomarker analysis and BDNF genotyping

At each timepoint, a 9-ml blood sample was collected, stored in ethylenediaminetetraacetic acid tubes, and centrifuged at 1069 × *g* for 10 min at 4* °C*. The plasma and buffy coat samples were stored in a − 80* °C* freezer prior to analysis. Plasma levels of IFN-ƴ, TNF-α, GM-CSF, IL-2, IL-4, IL-6, IL-8, and IL-10 were analyzed using the multiplexed immunoassay (Bioplex Human Cytokine 9-Plex Panel, Biorad). Cytokines with undetectable levels based on assay sensitivity were considered as 0 pg/ml. Plasma BDNF was assessed using published ELISA techniques. Biomarkers with > 80% of values that were below the lower limit of quantification were excluded from the final analysis [20]. As a possible confounder of plasma BDNF levels, the *BDNF* Val66Met (rs6265) polymorphism in the *BDNF* gene was genotyped from genomic DNA isolated from the buffy coat [43, 44]. Detailed procedures can be found in the Supplementary Methods.

### Primary and secondary outcomes

The primary outcome of this analysis is the association of plasma cytokine and BDNF levels with changes in objective and self-perceived cognition among AYAC during and post-chemotherapy in comparison to NC. As a secondary outcome, we also evaluate associations between post-baseline cytokine and BDNF levels.

### Statistical analysis

To assess differences in sociodemographic characteristics between AYAC and NC, categorical variables were assessed with Chi-square test or Fisher's exact test, and continuous variables with t-test or Mann–Whitney U test. Prevalence of post-baseline cognitive impairment (categorical) was calculated as the number of timepoints with cognitive impairment divided by the total number of timepoints within each category (NC, AYAC – active chemotherapy, AYAC – post-chemotherapy) with 95% confidence intervals (CIs) using the Wilson score method.

Differences in biomarker levels between groups (AYAC stratified by chemotherapy status vs. NC) and relative to baseline within each group were evaluated using multivariate regression analyses with log-transformed plasma biomarker levels as the outcome. Given that AYAC could contribute multiple post-baseline observations, models including AYAC were linear mixed models (LMM) with random intercepts to account for individual-specific effects. Models were adjusted for sociodemographic variables (age, sex assigned at birth, race/ethnicity, marital status, and education years), with models for BDNF also adjusted for BDNF Val66Met genotype [43, 44].

To assess associations between biomarkers with cognition, LMM analyses were first performed with interaction terms between biomarkers and group (NC, AYAC during active chemotherapy, AYAC post-chemotherapy) in predicting cognitive outcomes. Models were adjusted for baseline cognition, sociodemographic variables (as previously described), fatigue, and psychological distress [36, 37]. Biomarker-cognition trends were further elucidated in independent models for NC (linear regression) and AYAC (LMM) using Akaike Information Criterion (AIC)-guided model building. Briefly, all models were adjusted for baseline cognition, and the combination of biomarkers and confounders with the lowest AIC was selected. AYAC models were additionally adjusted for chemotherapy status, with interaction terms with biomarkers incorporated if indicated by AIC.

Post-baseline associations between cytokines and BDNF were analyzed according to the same procedures as biomarker-cognition associations, but with log-transformed plasma BDNF levels replacing cognition as the outcome and BDNF excluded from the list of biomarkers. Models were adjusted for sociodemographic variables, baseline BDNF levels, and BDNF Val66Met genotype.

Considering known problems with attrition and subsequent missing data in clinical studies of AYAC [45, 46] the LMM approach also allowed for the inclusion of all datapoints, with the aim of preventing potential selection bias resulting from complete case analysis. We also compared the characteristics of patients with biomarker data and at least one cognitive outcome versus those with cognition data only via univariate mixed effects logistic regression analyses with individuals as random intercepts.

All statistical analyses were conducted in R version 4.4 and tested at *p* < 0.05 [47]. Adjustment of multiple testing was not performed due to the exploratory nature of the analysis.

## Results

### Participant characteristics

After excluding nine participants who did not receive chemotherapy at any point during the study and seven NC who were lost to follow-up, 66 AYAC and 111 NC were included in the analysis (Table 1**, **Supplementary Fig. 1). There were more Malay (17% vs 2%) and fewer Indian (6% vs. 19%) participants in the AYAC group compared to the controls (*p* < 0.001). The AYAC included in this analysis were less educated (median years of education 16 vs. 17, *p* < 0.001) and slightly older (median age 34 vs. 32, *p* = 0.04) than NC. Most AYAC had a diagnosis of head and neck (24%) or breast (21%) cancer and received chemotherapies including platinum agents (68%), anthracyclines (29%), taxanes (26%), or methotrexate (5%). Forty-one AYAC contributed 51 post-baseline assessments during active chemotherapy, which were on average 4.4 months from baseline (SD = 2.8). Fifty-nine AYAC contributed 168 post-baseline assessments that were post-chemotherapy, which were on average 10.7 months from baseline (SD = 5.4). Sociodemographic and clinical characteristics were not significantly different among AYAC assessments with biomarker and cognition data compared to those with only cognition data, aside from AYAC in the Other race/ethnicity category having lower odds of providing biomarker data (Supplementary Table 1).

**Table 1:** Participant demographic and clinical characteristics

	AYAC (*N* = 66)	NC (*N* = 111)	***p***-value^a^
**Demographic Characteristics**			
Age in years, median (IQR)	34 (29, 37)	32 (28,35)	0.04
Sex assigned at birth, n (%)			
Male	25 (38%)	38 (34%)	0.74
Female	41 (62%)	73 (66%)	
Ethnicity, n (%)			
Chinese	46 (70%)	83 (75%)	< 0.001
Malay	11 (17%)	2 (2%)	
Indian	4 (6%)	21 (19%)	
Others^b^	5 (7%)	5 (4%)	
Marital status, n (%)			
Never married	24 (36%)	63 (57%)	0.06
Married	39 (59%)	46 (41%)	
Divorced	3 (5%)	1 (1%)	
Widowed	0 (0%)	1 (1%)	
Years of education, median (IQR)	16 (12, 17)	17 (16,19)	< 0.001
**Clinical Characteristics**			
Diagnosis, n (%)			
Breast	14 (21%)		
Head and neck	16 (24%)		
Gynecological	13 (20%)		
Lymphoma	9 (14%)		
Testicular	6 (9%)		
Sarcoma	3 (5%)		
Lung	2 (3%)		
Colorectal	2 (3%)		
Esophageal	1 (2%)		
Treatment Received, n (%)^c^			
Chemotherapies			
Anthracyclines	19 (29%)		
Taxanes	17 (26%)		
Platinum	45 (68%)		
Methotrexate	3 (5%)		
Radiation	36 (55%)		
Hormonal	16 (24%)		

*AYAC *adolescent and young adult cancer patients, *NC* non-cancer controls

^a^*p*-values for categorical variables were obtained using Chi-square test or Fisher's exact test, and continuous variables with t-test or Mann–Whitney U test

^b^Includes 2 Filipinos, 2 Sikhs, and 1 Bhutanese patients, as well as 4 Filipinos and 1 Vietnamese NC

^c^Treatments received at any point during the follow-up period, so categories are not mutually exclusive

### CRCI prevalence

**Fig. 1:**
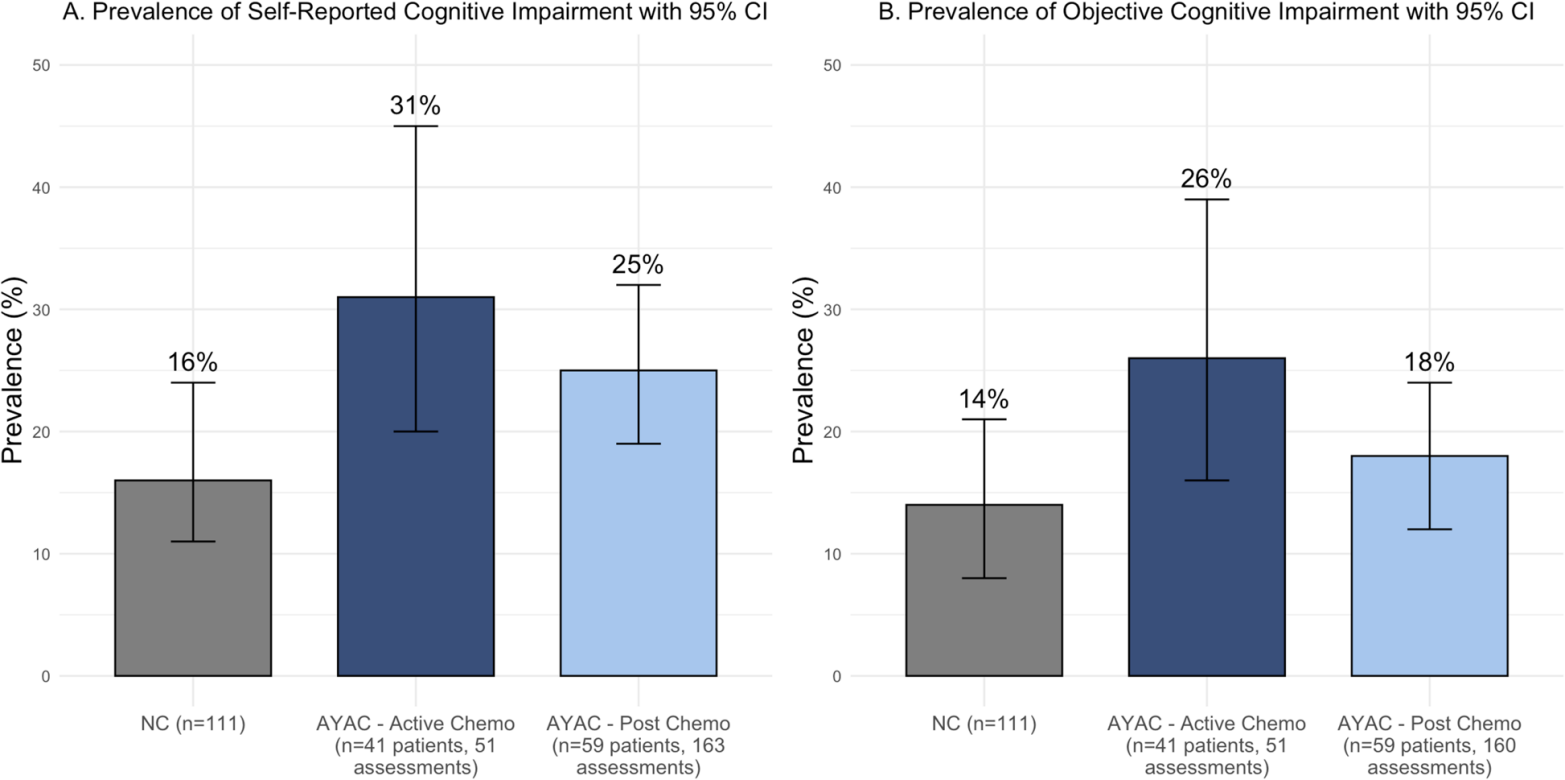
Prevalence of CRCI over time. **A** Prevalence of post-baseline self-perceived cognitive impairment among NC and AYAC (by chemotherapy status) with a 95% confidence interval. Self-perceived cognitive impairment is defined as a 10.6-point decline in FACT-Cog total score from baseline. **B** Prevalence of post-baseline objective cognitive impairment among NC and AYAC (by active chemotherapy status) with a 95% confidence interval over a 12-month period. Objective cognitive impairment is defined as a clinically significant decline (RCI < −1.96) in ≥ 1 cognitive domain(s) assessed by CANTAB. *AYAC* = adolescent and young adult cancer patients; *NC* = non-cancer controls

The prevalence of self-perceived cognitive impairment among AYAC undergoing chemotherapy and post-chemotherapy was 31% (95% CI 20 to 45%) and 25% (95% CI 19 to 32%) respectively, compared to 16% (95% CI 11 to 24%) for NC (Fig. 1A). We observed similar but less pronounced trends for objective cognitive impairment, with prevalence at 26% (95% 16 to 39%) for AYAC on active chemotherapy, 18% (95% CI 12 to 24%) for AYAC post-chemotherapy, and 14% (95% CI 8 to 21%) for NC (Fig. 1B). Cognitive domains most likely to show significant objective cognitive decline in AYAC both during and post-chemotherapy were memory, response speed, and attention (Supplementary Table 2).

### Plasma biomarker levels

**Fig. 2:**
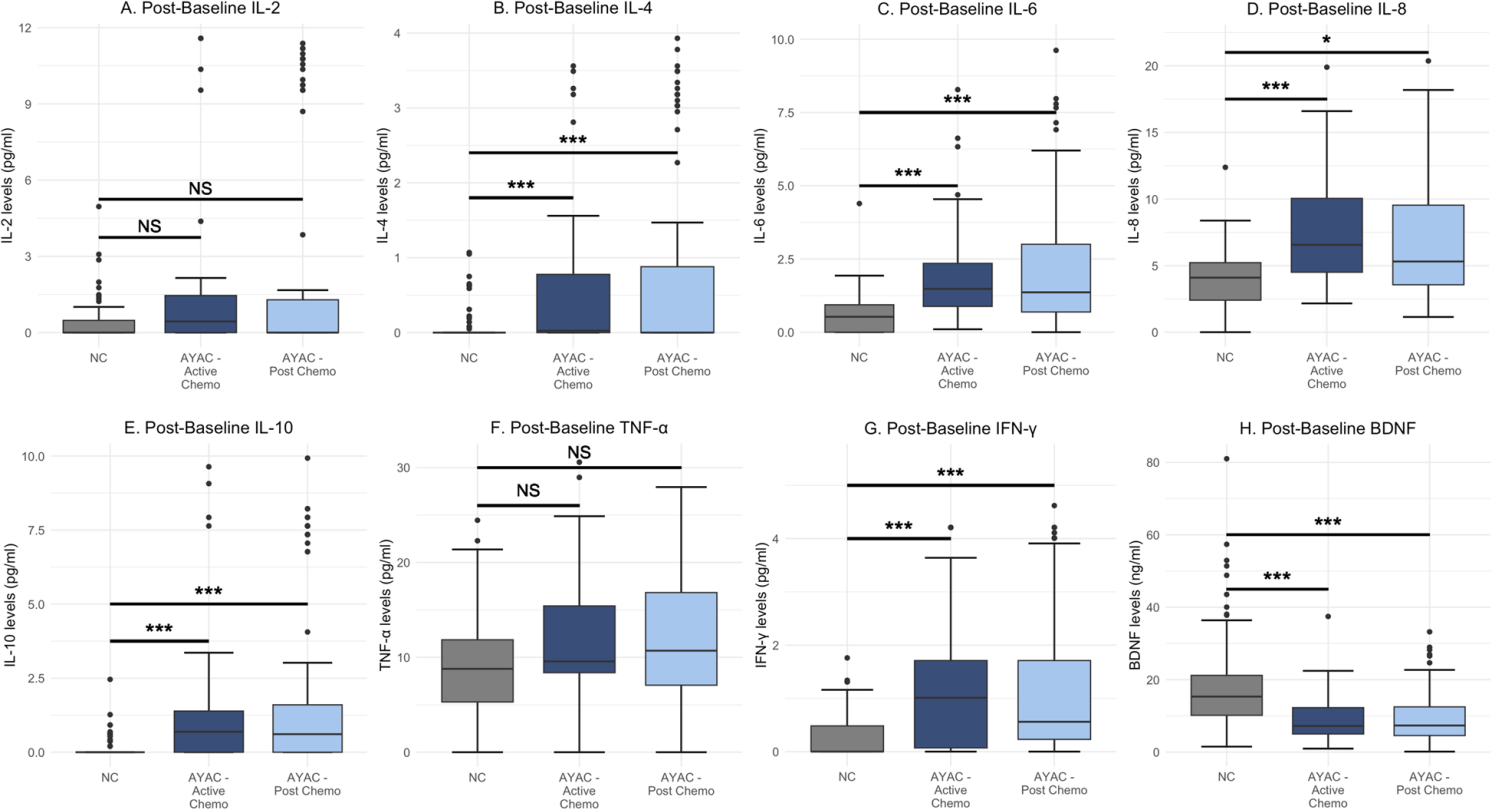
Post-baseline levels of cytokines and BDNF in AYAC compared to NC. Plasma levels of (**A**) IL-2, (**B**) IL-4, (**C**) IL-6, (**D**) IL-8, (**E**) IL-10, (**F**) TNF-α, (**G**) IFN-γ, and (**H**) BDNF in NC at follow-up (6 months post-baseline) and AYAC (follow-up timepoints stratified by chemotherapy status). The *p*-values denoted with asterisks represent significant differences from NC computed from linear mixed models with log-transformed levels as the outcome and adjusted for sociodemographic variables with individuals as random intercepts. *p < 0.05, **p < 0.01, ***p < 0.001. *AYAC* = adolescent and young adult cancer patients; *NC* = non-cancer controls; *IL* = interleukin; *TNF-α* = tumor necrosis factor alpha; *IFN-γ* = interferon gamma; *BDNF* = brain-derived neurotrophic factor; *NS = *not significant

GM-CSF was excluded from analysis due to > 80% of samples having undetectable plasma levels. Among AYAC, median plasma BDNF levels (ng/mL) post-chemotherapy were lower than baseline (7.35 vs. 10.56, *p* = 0.008) (Table 2A). Unexpectedly, we observed alterations in biomarker levels at follow-up among NC (Table 2B). Compared to baseline, NC had higher IL-2 (*p* = 0.01) and lower IL-10, IFN-γ, and BDNF plasma levels post-baseline (all *p* < 0.001). Compared to NC, AYAC had lower post-baseline plasma BDNF and elevated levels of IL-4, IL-6, IL-8, IL-10, and IFN-γ both during and post-chemotherapy (Fig. 2, all *p* < 0.05).

**Table 2:** Plasma levels of cytokines and BDNF for (A) AYAC at baseline and follow-up timepoints when receiving chemotherapy and post-chemotherapy; and (B) NC at baseline and 6 months post-baseline. Plasma levels are represented as median (IQR) in pg/mL for IL-2, IL-4, IL-6, IL-8, IL-10, TNF-α, and IFN-γ, and ng/mL for BDNF

**(A) AYAC**	**Baseline (*n*** **= 55)**	**Active Chemotherapy (*n*** **= 35 patients, 40 assessments)**	**Post Chemotherapy (*n*** **= 45 patients, 97 assessments)**
**IL-2 (pg/mL)**	0.00 (0.00, 1.21)	0.44 (0.00, 1.46)	0.00 (0.00, 1.29)
**IL-4 (pg/mL)**	0.00 (0.00, 0.63)	0.03 (0.00, 0.78)	0.00 (0.00, 0.88)
**IL-6 (pg/mL)**	1.90 (0.96, 3.32)	1.48 (0.92, 2.35)	1.36 (0.69, 3.00)
**IL-8 (pg/mL)**	5.56 (3.48, 11.07)	6.56 (4.50, 10.05)	5.31 (3.56, 9.54)
**IL-10 (pg/mL)**	0.53 (0.00, 1.62)	0.69 (0.00, 1.39)	0.61 (0.00, 1.60)
**TNF-α (pg/mL)**	9.43 (6.27, 15.24)	9.57 (8.38, 15.42)	10.70 (7.07, 16.83)
**IFN-γ (pg/mL)**	0.80 (0.32, 1.56)	1.01 (0.09, 1.79)	0.56 (0.23, 1.71)
**BDNF (ng/mL)**	10.56 (6.68, 14.98)	7.22 (5.00, 12.27)	7.35 (4.57, 12.48)**
**(B)** **NC**	**Baseline (*n*** **= 111)**	**6-months post-baseline (*n*** **= 108)**	
**IL-2 (pg/mL)**	0.00 (0.00, 0.12)	0.00 (0.00, 0.48)*	
**IL-4 (pg/mL)**	0.00 (0.00, 0.00)	0.00 (0.00, 0.00)	
**IL-6 (pg/mL)**	0.50 (0.00, 1.04)	0.53 (0.00, 0.94)	
**IL-8 (pg/mL)**	4.25 (2.62, 5.73)	4.10 (2.40, 5.22)	
**IL-10 (pg/mL)**	0.00 (0.00, 0.53)	0.00 (0.00, 0.00)***	
**TNF-α (pg/mL)**	9.27 (6.95, 12.04)	8.79 (5.30, 11.85)	
**IFN-γ (pg/mL)**	0.33 (0.00, 0.60)	0.00 (0.00, 0.482)***	
**BDNF (ng/mL)**	22.48 (16.13, 28.70)	15.32 (10.16, 21.19)***	

*AYAC* adolescent and young adult cancer patients, *NC* non-cancer controls, *IL* interleukin, *TNF-α* tumor necrosis factor alpha, *IFN-γ* interferon gamma, *BDNF *brain-derived neurotrophic factor

^*^p < 0.05, **p < 0.01, ***p < 0.001; *p*-values represent significant differences from baseline computed from linear mixed models with log-transformed levels as the outcome and adjusted for sociodemographic variables with individuals as random intercepts

### Relationships between biomarkers and cognition

#### Interactions between group (AYAC during active and post-chemotherapy vs. NC) and biomarkers in predicting cognition

**Fig. 3:**
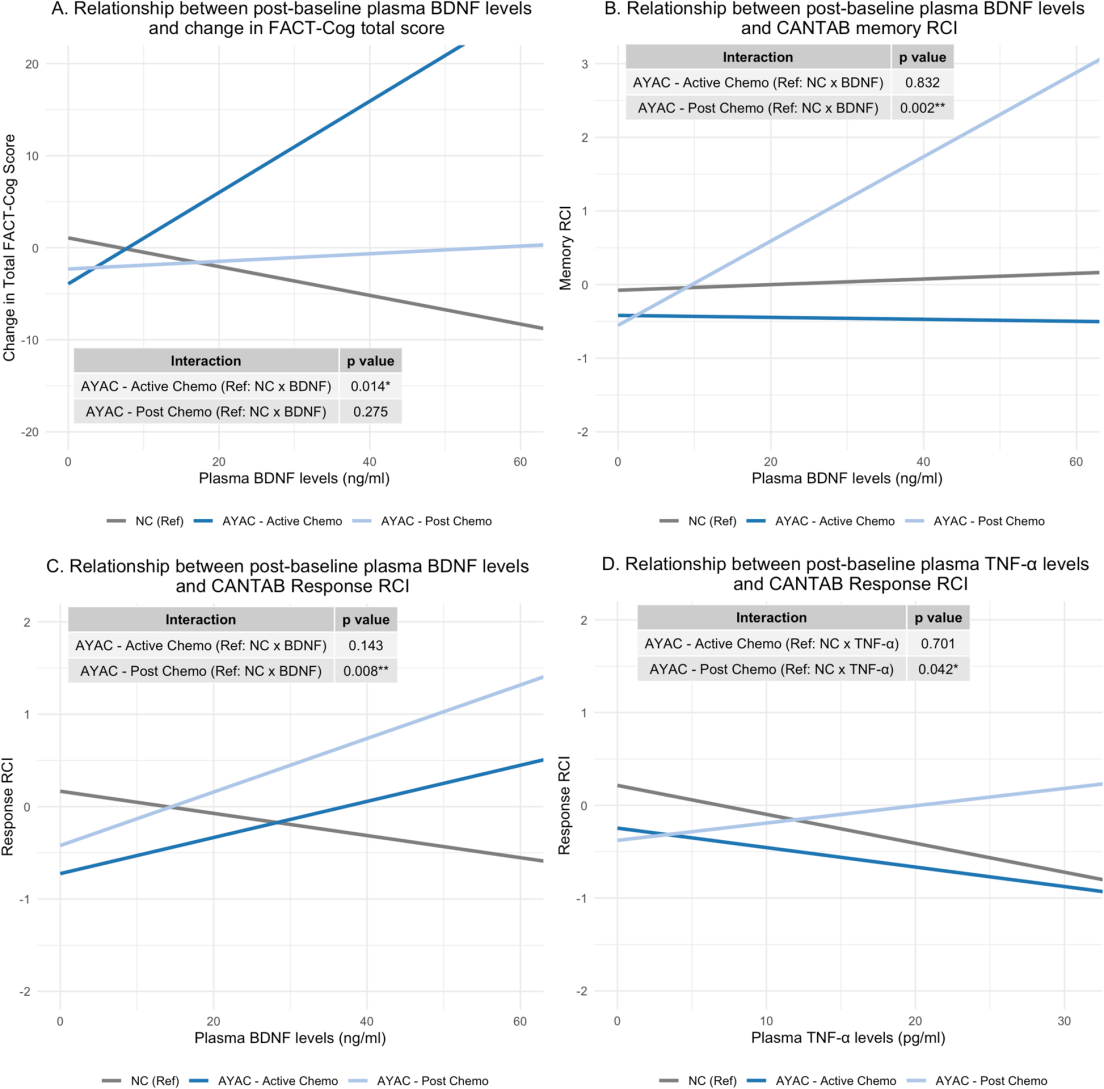
Biomarker-cognition associations among NC and AYAC. Significant interaction plots from linear mixed models investigating the associations of biomarkers with cognition in AYAC and NC. The analysis was performed with interaction terms for each biomarker and group (AYAC stratified by chemotherapy status vs. NC) in predicting cognition with adjustments for baseline cognition, sociodemographic variables, fatigue, and psychological distress. Lower change in total FACT-Cog score, memory RCI, and response RCI indicate cognitive decline. Median change in total FACT-Cog score = −0.5 (IQR = −9.0, 6; range = −79, 58). Median memory RCI = 0.13 (IQR = −0.37, 0.54; range = −6.24, 3.56). Median response RCI = −0.06 (IQR = −0.84, 0.6; range = −3.75, 2.68). *p < 0.05, **p < 0.01, ***p < 0.001. *AYAC* = adolescent and young adult cancer patients; *NC* = non-cancer controls; *TNF-α* = tumor necrosis factor alpha; *BDNF* = brain-derived neurotrophic factor; *RCI* = reliable change index; *FACT-Cog* = Functional Assessment of Cancer Therapy-Cognitive Function; *CANTAB* = Cambridge Neuropsychological Test Automated Battery

First, we investigated differences in post-baseline biomarker-cognition associations between NC and AYAC (stratified by chemotherapy type) using interaction terms (Fig. 3). There was a significant interaction between group and BDNF in predicting self-perceived cognition, indicating a positive BDNF-cognition association among AYAC on active chemotherapy but not NC (Fig. 3A, *p* = 0.014). There were also significant interactions between group and BDNF in predicting memory (*p* = 0.002) and response speed (*p* = 0.008), indicating positive BDNF-cognition associations among AYAC post-chemotherapy but not NC (Fig. 3B-C). A significant interaction between group with TNF-α in predicting response speed indicated a positive TNF-α -cognition association among AYAC post-chemotherapy but not NC (Fig. 3D, *p* = 0.042). All other interactions between biomarkers and group (AYAC stratified by chemotherapy status vs. NC) in predicting cognition were non-significant (Supplementary Figs. 2, 3, 4, 5, 6 and 7).

#### Biomarker-cognition associations in AYAC and NC subgroup models

In separated models for AYAC and NC, lower post-baseline IL-10 was significantly associated with worse self-perceived cognition among AYAC, while higher BDNF was significantly associated with worse self-perceived cognition among NC (Table 3, both *p* < 0.05). For objective cognition among AYAC, higher post-baseline IL-6 was significantly associated with worse multitasking, response speed, and attention, IL-8 with better multitasking, TNF-α with worse multitasking and better memory, and BDNF with better response speed and memory (Table 3, all *p* < 0.05). There was a significant interaction between chemotherapy status (active vs. post-chemotherapy) and BDNF in predicting memory, indicating a stronger relationship between BDNF levels and memory among AYAC post-chemotherapy. Among NC, TNF-α was significantly associated with worse multitasking and BDNF with better multitasking (*p* = 0.013 and 0.040, respectively).

**Table 3:** Statistically significant associations between cytokines and BDNF with change in cognition differ between AYAC and NC

**Biomarker**	**AYAC**^a^	**NC**^b^
IL-2	NS	NS
IL-4	NS	NS
IL-6	Worse multitasking (*p* = 0.026); worse response speed (*p* = 0.047), worse attention (*p* = 0.035)	NS
IL-8	Better multitasking (*p* = 0.004)	NS
IL-10	Better self-perceived cognition (*p* = 0.015)	NS
TNF-α	Worse multitasking (*p* = 0.025); better memory (*p* = 0.004)	Worse multitasking (*p* = 0.013)
IFN-γ	NS	NS
BDNF	Better memory (*p* = 0.002); better response speed (*p* = 0.028)	Worse self-perceived cognition (*p* = 0.040); better multitasking (*p* = 0.040)

*AYAC* adolescent and young adult cancer patients, *NC* non-cancer controls, *IL* interleukin, *TNF-α* tumor necrosis factor alpha, *IFN-γ* interferon gamma, *BDNF* brain-derived neurotrophic factor, *NS* not significant

^a^ *p*-values for AYAC were computed from linear mixed models with individuals as random intercepts, adjusted for baseline cognition and chemotherapy status, with confounders and biomarkers incorporated using a model-building approach

^b^*p*-values for NC were computed from linear regression models, adjusted for baseline cognition, with confounders and biomarkers incorporated using a model-building approach

### Relationships between cytokines and BDNF

There were no significant interactions between group (AYAC stratified by chemotherapy status vs. NC) and cytokines in predicting post-baseline BDNF levels (Supplementary Fig. 8). In the separated models for AYAC and NC, higher IL-4 and lower IL-10 were significantly associated with higher BDNF among AYAC (*p* = 0.003 and 0.002, respectively) (Table 4). Lower IFN-γ was significantly associated with higher BDNF levels among NC (*p* = 0.016). Higher TNF-α was significantly associated with higher BDNF levels in both AYAC and NC (*p* = 0.006 and 0.009, respectively). Among AYAC, these trends did not significantly differ based on chemotherapy status (active vs. post-chemotherapy), as indicated by a lack of significant interaction terms.

**Table 4:** Statistically significant associations between post-baseline cytokine and BDNF levels differ between AYAC and NC

Biomarker	AYAC^a^	NC^b^
IL-2	NS	NS
IL-4	Higher BDNF (*p* = 0.003)	NS
IL-6	NS	NS
IL-8	NS	NS
IL-10	Lower BDNF (*p* = 0.002)	NS
TNF-α	Higher BDNF (*p* = 0.006)	Higher BDNF (*p* = 0.009)
IFN-γ	NS	Lower BDNF (*p* = 0.016)

*AYAC* adolescent and young adult cancer patients, *NC* non-cancer controls, *IL *interleukin, *TNF-α *tumor necrosis factor alpha, *IFN-γ *interferon gamma, *BDNF *brain-derived neurotrophic factor, *NS* not significant

^a^ *p*-values for AYAC were computed from linear mixed models with individuals as random intercepts, adjusted for baseline BDNF and chemotherapy status, with confounders and biomarkers incorporated using a model-building approach

^b^*p*-values for NC were computed from linear regression models, adjusted for baseline cognition, with confounders and biomarkers incorporated using a model-building approach

## Discussion

In our longitudinal observational study of AYAC and NC, we found that lower plasma BDNF and dysregulated plasma IL-6, IL-8, IL-10, and TNF-α were associated with chemotherapy-induced declines in self-perceived and objective cognition among AYAC. Few of these associations were also observed in NC. We also found that cytokines were predictive of BDNF levels among both AYAC and NC, although patterns differed between the two groups. Notably, these findings provide novel insight on relationships between inflammation, BDNF, and CRCI that are unique to the cancer population by limiting age-related confounders.

Previous studies linking plasma cytokine and/or BDNF levels to CRCI have focused mainly on breast cancer patients and lack a relevant comparison group [8, 11, 17, 20, 48, 49]. Our inclusion of NCs demonstrates a robust approach to identifying biomarkers associated with cognitive impairment that are specific to cancer patients, providing clearer insight into possible CRCI mechanisms. Unexpectedly, our NC group had statistically significantly altered plasma levels of IL-2, IL-10, IFN-γ, and BDNF at 6-months follow-up compared to baseline, while among AYAC only a decline in BDNF levels was observed during follow-ups post-chemotherapy. While NCs lack a cancer diagnosis, there might be other unknown health conditions and/or lifestyle factors associated with circulating cytokine and BDNF levels that may have contributed to these observed changes [50–52]. Our study timeline of 2018 to 2023 also coincided with the COVID-19 pandemic and lockdown period, whereby a decline in mental health and physical activity was observed among adolescents, which may have an impact on the biomarkers we are investigating [50–53]. Despite these observed longitudinal changes in NC, the majority of associations between biomarkers and cognition were unique to AYAC.

Given that AYAC are being treated with different types of chemotherapy for their respective cancer conditions, our study has taken a novel approach to investigate the impact of treatment on the cognitive outcomes. Along with the heterogeneity of treatment regimens among AYAC and the evidence that cognitive impairment persists up to one year after diagnosis [24], we analyzed how biomarker-cognition trends varied during and post-chemotherapy. AYAC both during and post-chemotherapy had higher rates of self-perceived and objective cognitive impairment, lower plasma BDNF, and elevated IL-4, IL-6, IL-8, IL-10, and IFN-γ compared to NC during follow-up. Our findings suggest that not only do cognitive decline and altered biomarker trends remain post-chemotherapy, but biomarker-cognition trends may vary depending on treatment status. For instance, interaction analyses revealed that the association between BDNF with better self-perceived cognition was unique to AYAC during chemotherapy, while the association between TNF-α and response speed was positive among AYAC post-chemotherapy but negative during chemotherapy and among AYAC. It is important to note that a longitudinal study of breast cancer patients found that associations between cytokines and cognition remained up to two years after chemotherapy initiation, and that these relationships varied over time [11]. With an AYAC cohort, our findings provide novel insight on how chemotherapy may drive long-term biomarker-cognition relationships independent of age-related cognitive decline. We observed relationships between inflammation and BDNF with CRCI to be dependent on current chemotherapy exposure status, and clinical interventions should target both current patients and survivors.

Our findings add to the existing literature that have demonstrated links between plasma cytokine and/or BDNF levels with CRCI. Similar to our findings, dysregulated plasma levels of IL-6, IL-8 and TNF-α have also been observed in patients with CRCI in breast cancer and hematological malignancies [6–10]. These observations underscore the potential of using cytokine levels to identify and screen for cancer patients who are more likely to have CRCI for additional evaluation using patient-reported tools and neuropsychological testing. Given the time and expertise needed for comprehensive cognitive assessment, employing biomarkers as an initial screening tool for CRCI during routine follow-up visits may represent a more efficient use of resources. Nevertheless, further understanding of the relationship between biomarkers and CRCI is required before implementation in the clinical setting. Although positive associations between plasma IL-6 levels and CRCI have been noted in different studies, cognitive domains that were observed to be affected have been inconsistent [8, 10]. IL-1β and IL-4 have also been linked to cognitive performance among cancer patients in some studies, but no associations were found in our study [6, 8]. Heterogeneity between study populations, particularly pertaining to the presence of age-related confounders, could be contributing to these inconsistencies. By limiting these confounders, our approach provides strong evidence of the relationship between the identified cytokines and CRCI. These inconsistencies may also be a result of the cross-linking and cascading nature of cytokine-mediated neuroinflammatory pathways in the human body, which suggests that the use of cytokine profiles rather than the level of a specific cytokine as an indicator of CRCI risk may be more appropriate [54].

Furthermore, our proposed approach of considering cytokines in conjunction with BDNF as a mediator can facilitate improved usage of these markers. Our results suggest that cytokine levels are significantly associated with observed BDNF levels in our patient population. Although we are unable to establish causality with these associations, preclinical studies implicate a bidirectional relationship between cytokines and BDNF. In vivo studies have shown a decrease in BDNF expression as a result of inflammatory stimulation by lipopolysaccharide [18, 19]. Conversely, BDNF treatment in primary microglia was shown to reverse the release of proinflammatory cytokines IL-6 and TNF-α, indicating an anti-inflammatory effect [15]. During the acute phase of treatment, if dysregulated inflammation is observed through cytokine measurements, BDNF levels could then be evaluated to establish if they have decreased or are lower than expected. Lower BDNF levels would demonstrate potential neurotoxic effects of inflammation, and cognitive toxicities may be significantly more likely [55].

By underscoring the role of neuroinflammation as an important mechanistic pathway in CRCI, these findings further highlight the critical need to expand therapeutic strategies targeting this pathway [56–58]. While pharmacological options for addressing CRCI remain limited and largely untested in clinical settings [56, 58], non-pharmacological interventions such as physical activity have demonstrated effectiveness in increasing BDNF levels and may offer significant benefit [59–61]. BDNF augmentation via the glutamate blocker riluzole has been shown to reverse doxorubicin-induced CRCI in mice, but there are currently no studies on clinical efficacy [62]. The persistence of CRCI and biomarker dysregulation beyond the chemotherapy phase suggests that interventions should begin during treatment and continue into survivorship to provide sustained support. Proactive monitoring of inflammatory cytokines and neurotrophic markers such as BDNF could enable early identification of at-risk individuals and guide personalized interventions to mitigate long-term cognitive deficits. Moreover, the susceptibility of AYAC to conditions such as depression, anxiety, and substance use [63–66] further predisposes them to elevated inflammatory cytokines and reduced BDNF, placing this population at heightened risk for prolonged CRCI. By instituting early, biomarker-informed interventions tailored to the unique vulnerabilities of AYAC, clinicians can address the burden of chronic cognitive impairment, ultimately enhancing quality of life and functional independence throughout survivorship.

A key strength of our analysis is our choice of evaluating biomarker associations with cognition in a AYA cohort of cancer and non-cancer participants. Unlike in other more homogenous cancer cohorts, any associations found unique among participants with cancer are attributable to CRCI with certainty given the low risk of age-related neurocognitive problems among AYAs. This poses a difficulty with recruitment given the relatively low prevalence of AYAC patients diagnosed within a general cancer population. Recruitment strategy was thus focused on meeting the pre-determined age ranges across different sites without restrictions on types of cancer and treatments. Consequently, our recruited patients were highly heterogeneous, leading to challenges to ensure both internal and external validity of our findings. Future large-scale, multi-site studies could evaluate subgroup differences based on gender, further stratified age ranges, and cancer diagnosis. Rather, our analyses centered on redefining assessment timepoints relative to chemotherapy exposure—specifically anthracyclines, taxanes, platinum agents, and methotrexate—due to their known neurotoxic potential and associated cognitive decline risks. Such an approach not only improves clinical interpretation of our findings but also sets the groundwork for future AYAC research studies to adopt when conducting cancer survivorship and supportive care studies.

## Conclusions

This study establishes that interactions between dysregulated cytokine signaling and altered BDNF expression may play a role in CRCI among AYAC. Several observed associations were unique to the cancer condition as well as varied depending on whether patients were actively undergoing chemotherapy or > 30 days removed from their last dose, highlighting the need for intervention in both patient care and survivorship settings. As precision medicine becomes integrated into AYA patient care, plasma BDNF and cytokines may serve as important predictors of CRCI onset.

## Supplementary Information

Supplementary Material 1. This file contains additional details on methodology and supplementary figures and tables.

## Data Availability

The datasets used and/or analyzed during the current study are available from the corresponding author on reasonable request.

## Abbreviations

AYAC: Adolescent and young adult cancer patients
NC: Non-cancer controls
CRCI: Cancer-related cognitive impairment
IL: Interleukin
TNF-α: Tumor necrosis factor alpha
IFN-γ: Interferon gamma
BDNF: Brain-derived neurotrophic factor
RCI: Reliable change index
FACT-Cog: Functional Assessment of Cancer Therapy-Cognitive Function
CANTAB: Cambridge Neuropsychological Test Automated Battery
