# Supplementary material for: Brain-derived neurotrophic factor and cytokines as predictors of cognitive impairment in adolescent and young adult cancer patients receiving chemotherapy: a longitudinal study
Source: BMC Cancer. 2025 Jul 1;25:1045. doi: 10.1186/s12885-025-14430-3 (PMC12211463; doi:10.1186/s12885-025-14430-3)
Supplement: Supplementary file 1 — Supplementary Material 1. This file contains additional details on methodology and supplementary figures and tables. [file 12885_2025_14430_MOESM1_ESM.pdf]

Supplementary Materials

Supplementary Methods

Biomarker Analysis and BDNF Genotyping

Inflammatory cytokines were quantified using 50 µl of each plasma sample with the multiplexed immunoassay (Bioplex Human Cytokine 9-Plex Panel, Biorad) performed in duplicate with results expressed as pg/ml. The cytokine panel included interferon (IFN)-ɣ, tumor-necrosis factor (TNF-α), granulocyte-macrophage colony-stimulating factor (GM-CSF), interleukin (IL)-2, IL-4, IL-6, IL-8, and IL-10. Cytokines with undetectable levels in >80% of samples were excluded from analysis.

BDNF levels were quantified using 100 µl of plasma sample diluted 100-fold using a commercially available enzyme-linked immunosorbent assay (ELISA) kit (Biosensis BEK-2211-1P/2P, Australia) and performed in duplicate. The concentration of BDNF was calculated with four-parameter logistic regression followed by transformation to ng/ml.

Genomic DNA was isolated from the buffy coat using the QIAamp DNA Blood Mini Kit (Qiagen, Germany). Subsequently, the Val66Met polymorphism in BDNF gene was amplified using polymerase chain reaction (PCR) and genotyped using automated Sanger sequencing using a 3730xl DNA Analyzer (Applied Biosystems, USA).

Supplementary Figures and Tables

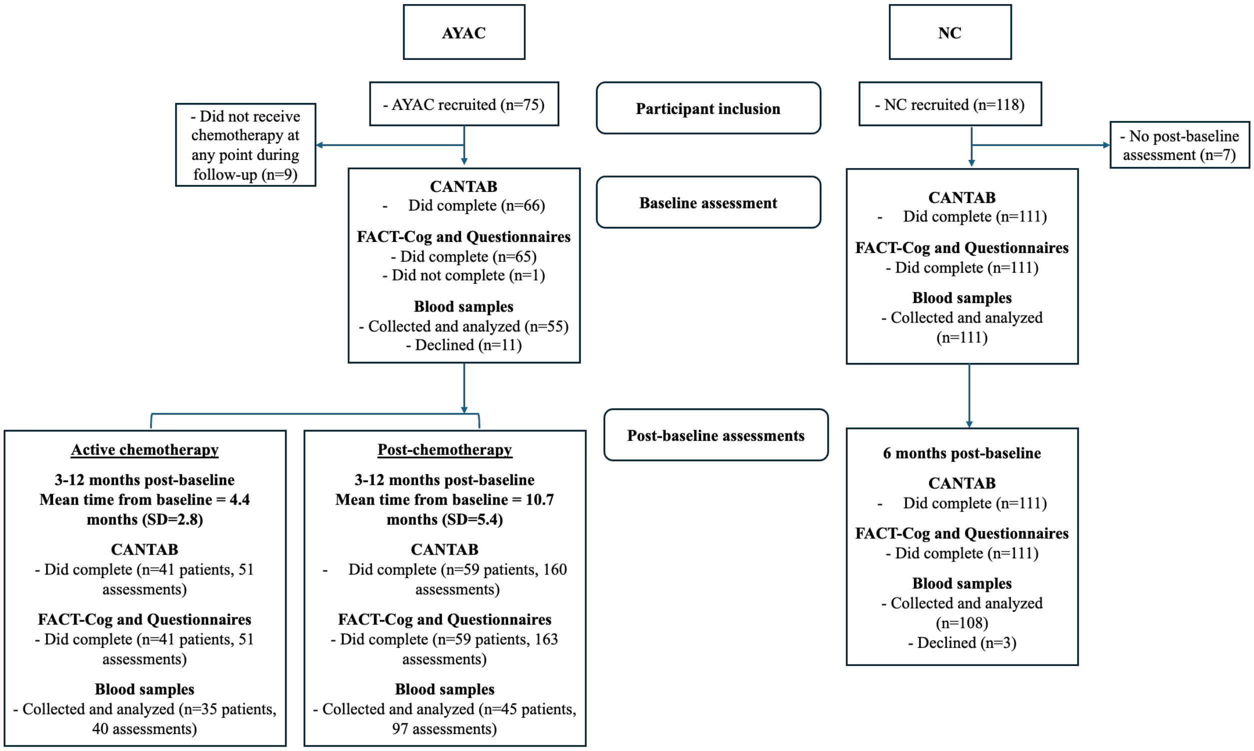

Supplementary Figure 1. Participant recruitment flowchart.
AYAC = adolescent and young adult cancer patients; *NC* = non-cancer controls; *CANTAB* = Cambridge Neuropsychological Test Automated Battery; *FACT-Cog* = Functional Assessment of Cancer Therapy-Cognitive Function version 3.

Supplementary Table 1. Univariate analyses comparing AYAC timepoints with and without biomarker data. This table compares AYAC characteristics among timepoints with at least one cognitive outcome that include biomarker data (vs. cognition data only), reported as odds ratios with 95% confidence intervals.

	**OR (95% CI)**	***p* value**[a]
**Demographic Characteristics**		
Age in years	1.03 (0.91 – 1.18)	0.615
Sex assigned at birth		
Female (Ref: Male)	1.55 (0.37 – 6.44)	0.546
Ethnicity		
Malay (Ref: Chinese)	0.33 (0.06 – 1.90)	0.214
Indian (Ref: Chinese)	0.08 (0.01 – 1.37)	0.082
Others (Ref: Chinese)	0.07 (0.01 – 0.87)	**0.038**
Marital status		
Married (Ref: Never married)	0.30 (0.07 – 1.29)	0.107
Divorced (Ref: Never married)	0.29 (0.01 – 7.85)	0.463
Years of education	1.07 (0.88 – 1.31)	0.485
**Clinical Characteristics**		
Diagnosis		
Breast	1.02 (0.18 – 5.79)	0.985
Head and neck	0.41 (0.08 – 1.99)	0.267
Gynecological	2.89 (0.49 – 17.00)	0.240
Lymphoma	3.89 (0.52 – 29.30)	0.186
Testicular	0.27 (0.03 – 2.97)	0.286
Sarcoma	0.16 (0.01 – 3.99)	0.264
Lung	4.69 (0.06 – 349.00)	0.482
Colorectal	0.80 (0.02 – 36.30)	0.909
Esophageal	1.02 (0.01 – 204.00)	0.993
Treatment Received		
Chemotherapies		
Anthracyclines	0.58 (0.13 – 2.70)	0.491
Taxanes	3.37 (0.63 – 18.10)	0.157
Platinum	0.85 (0.19 – 3.77)	0.827
Methotrexate	6.30 (0.18 – 224.00)	0.312
Radiation	0.36 (0.09 – 1.45)	0.150
Hormonal	1.23 (0.23 – 6.50)	0.807

[a] *p* values retrieved from univariate mixed effects logistic regression analyses with individuals as random intercepts, comparing assessments with biomarker data and at least one cognitive outcome (n=192 assessments) vs. cognition data only (n=93 assessments). Significant *p* values (<0.05) are indicated in bold.
OR = odds ratio, *CI* = confidence interval.

Supplementary Table 2. Prevalence of objective impairment stratified by domain. Prevalence of post-baseline objective cognitive decline among NC and AYAC (by chemotherapy status) with a 95% confidence interval over a 12-month period.

Objective cognitive impairment prevalence, % (95% CI) [a]	NC (n=111)	AYAC – Active chemotherapy (n=41 patients, 51 assessments)	AYAC – Post chemotherapy (n=59 patients, 160 assessments)
**Multitasking**	2.7% (0.9, 7.6)	2.0% (0.3, 10.3)	2.5% (1.0, 6.3)
**Memory**	5.4% (2.5, 11.3)	5.9% (2.0, 15.9)	6.9% (3.9, 11.9)
**Response speed**	2.7% (0.9, 7.6)	11.8% (5.5, 23.4)	5.6% (3.0, 10.3)
**Executive function**	0% (0, 0)	0% (0, 0.1)	0% (0, 0)
**Attention**	3.6% (1.4, 8.9)	9.8% (4.3, 20.1)	4.4% (2.1, 8.8)

[a] Objective cognitive impairment in each domain is defined as a clinically significant decline (RCI < -1.96) assessed by CANTAB.

AYAC = adolescent and young adult cancer patients; *NC* = non-cancer controls.

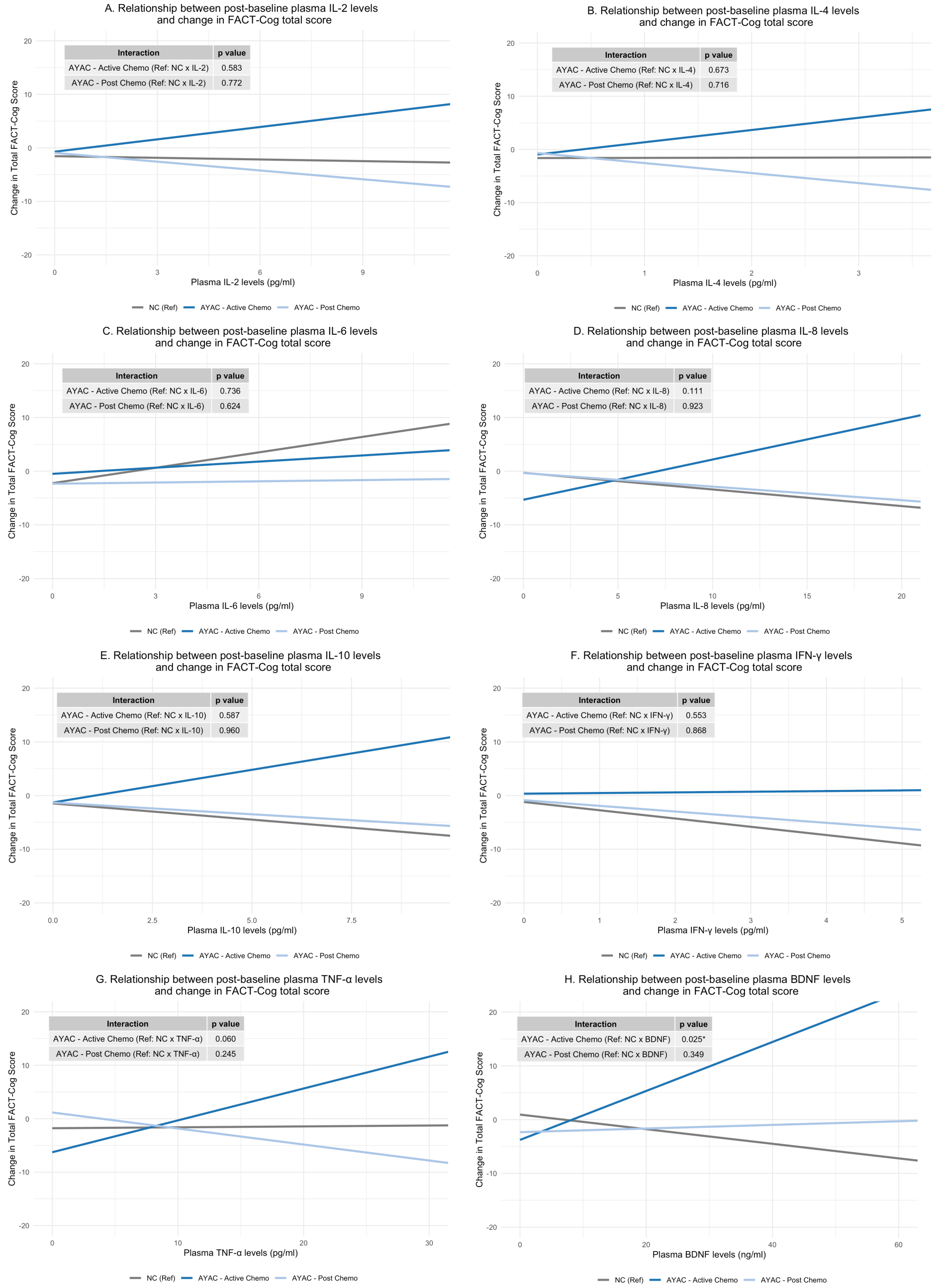

Supplementary Figure 2. Associations between biomarkers with change in self-perceived cognition among NC and AYAC. Interaction plots from linear mixed models investigating the associations of (A) IL-2, (B) IL-4, (C) IL-6, (D) IL-8, (E) IL-10, (F) IFN-γ, (G) TNF-α, and (H) BDNF with change in self-perceived cognition (total FACT-Cog score) in AYAC and NC. The analysis was performed with interaction terms for each biomarker and group (AYAC stratified by chemotherapy status vs. NC) in predicting cognition with adjustments for baseline cognition, sociodemographic variables, fatigue, and psychological distress. A negative change in total FACT-Cog score indicates cognitive decline from baseline. *$p < 0.05$.

AYAC = adolescent and young adult cancer patients; *NC* = non-cancer controls; *IL* = interleukin; *TNF-α* = tumor necrosis factor alpha; *IFN-γ* = interferon gamma; *BDNF* = brain-derived neurotrophic factor; *FACT-Cog* = Functional Assessment of Cancer Therapy-Cognitive Function version 3.

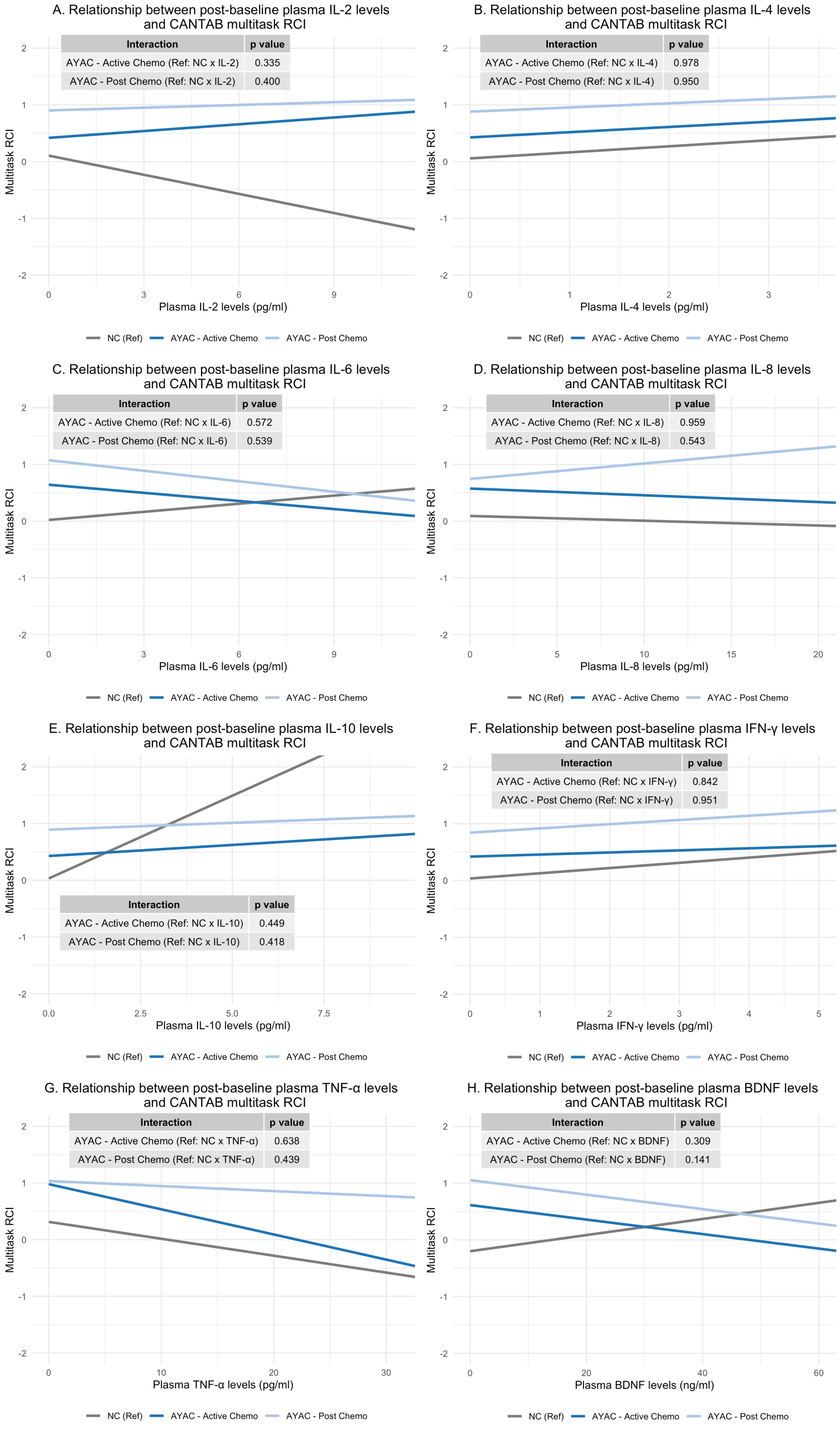

Supplementary Figure 3. Associations between biomarkers with multitasking among NC and AYAC. Interaction plots from linear mixed models investigating the associations of (A) IL-2, (B) IL-4, (C) IL-6, (D) IL-8, (E) IL-10, (F) IFN-γ, (G) TNF-α, and (H) BDNF with CANTAB multitasking RCI in AYAC and NC. The analysis was performed with interaction terms for each biomarker and group (AYAC stratified by chemotherapy status vs. NC) in predicting cognition with adjustments for baseline cognition, sociodemographic variables, fatigue, and psychological distress. A negative RCI indicates cognitive decline from baseline.
AYAC = adolescent and young adult cancer patients; *NC* = non-cancer controls; *IL* = interleukin; *TNF-α* = tumor necrosis factor alpha; *IFN-γ* = interferon gamma; *BDNF* = brain-derived neurotrophic factor; *CANTAB* = Cambridge Neuropsychological Test Automated Battery; *RCI* = reliable change index.

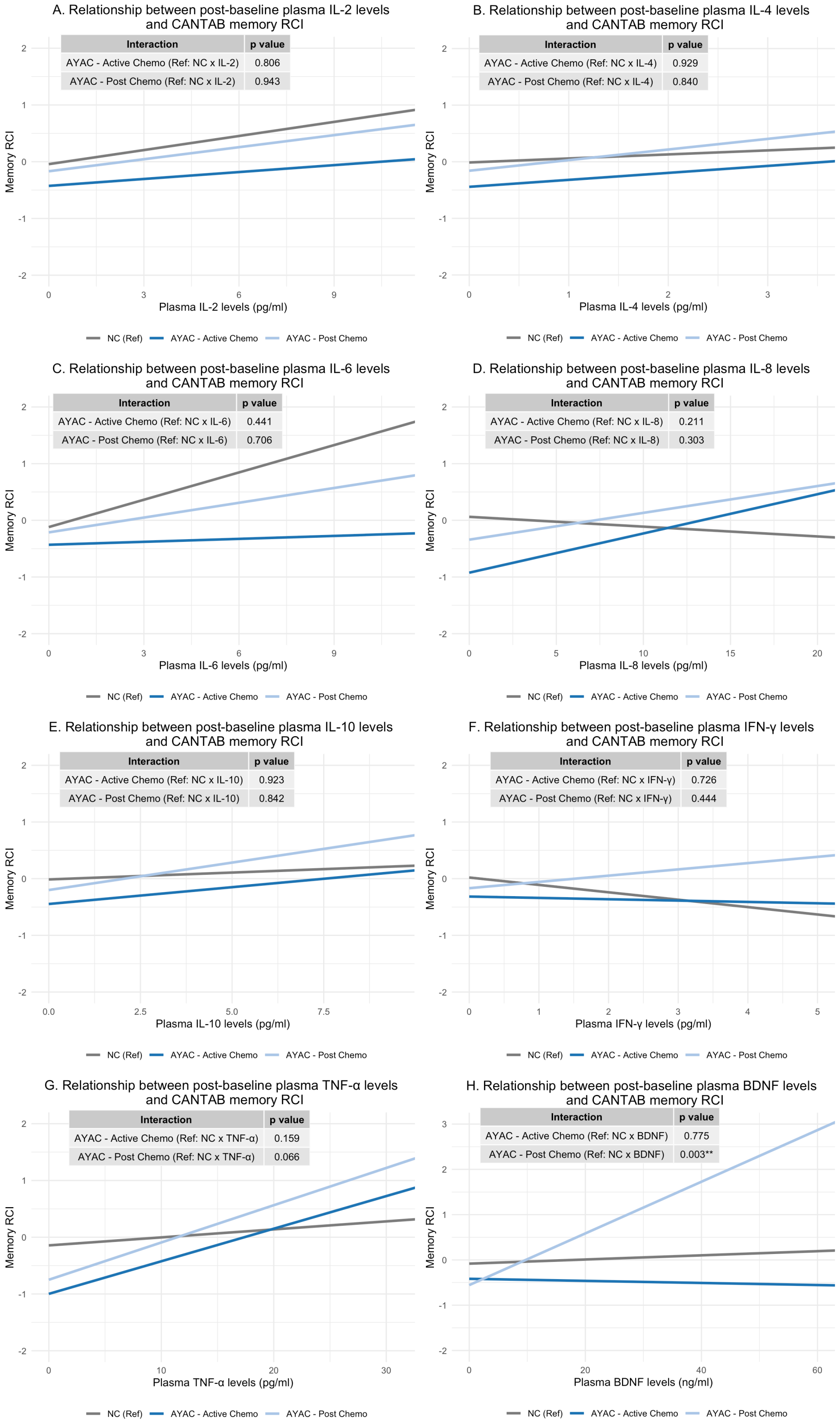

Supplementary Figure 4. Associations between biomarkers with memory among NC and AYAC. Interaction plots from linear mixed models investigating the associations of (A) IL-2, (B) IL-4, (C) IL-6, (D) IL-8, (E) IL-10, (F) IFN-γ, (G) TNF-α, and (H) BDNF with CANTAB memory RCI in AYAC and NC. The analysis was performed with interaction terms for each biomarker and group (AYAC stratified by chemotherapy status vs. NC) in predicting cognition with adjustments for baseline cognition, sociodemographic variables, fatigue, and psychological distress. A negative RCI indicates cognitive decline from baseline. ***p* < 0.01.
AYAC = adolescent and young adult cancer patients; *NC* = non-cancer controls; *IL* = interleukin; *TNF-α* = tumor necrosis factor alpha; *IFN-γ* = interferon gamma; *BDNF* = brain-derived neurotrophic factor; *CANTAB* = Cambridge Neuropsychological Test Automated Battery; *RCI* = reliable change index.

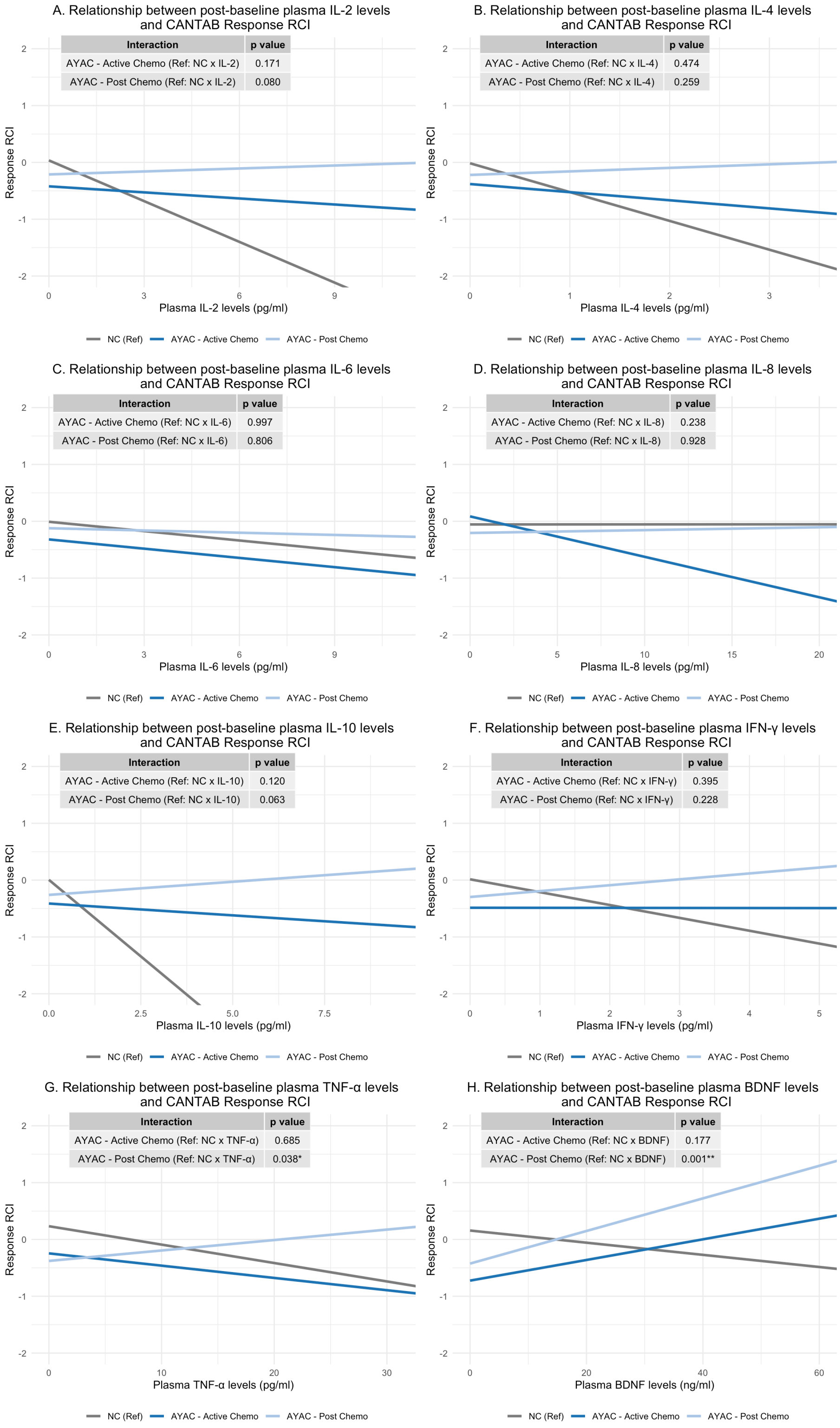

Supplementary Figure 5. Associations between biomarkers with response speed among NC and AYAC. Interaction plots from linear mixed models investigating the associations of (A) IL-2, (B) IL-4, (C) IL-6, (D) IL-8, (E) IL-10, (F) IFN-γ, (G) TNF-α, and (H) BDNF with CANTAB response RCI in AYAC and NC. The analysis was performed with interaction terms for each biomarker and group (AYAC stratified by chemotherapy status vs. NC) in predicting cognition with adjustments for baseline cognition, sociodemographic variables, fatigue, and psychological distress. A negative RCI indicates cognitive decline from baseline. $*p < 0.05$, $**p < 0.01$.

AYAC = adolescent and young adult cancer patients; *NC* = non-cancer controls; *IL* = interleukin; *TNF-α* = tumor necrosis factor alpha; *IFN-γ* = interferon gamma; *BDNF* = brain-derived neurotrophic factor; *CANTAB* = Cambridge Neuropsychological Test Automated Battery; *RCI* = reliable change index.

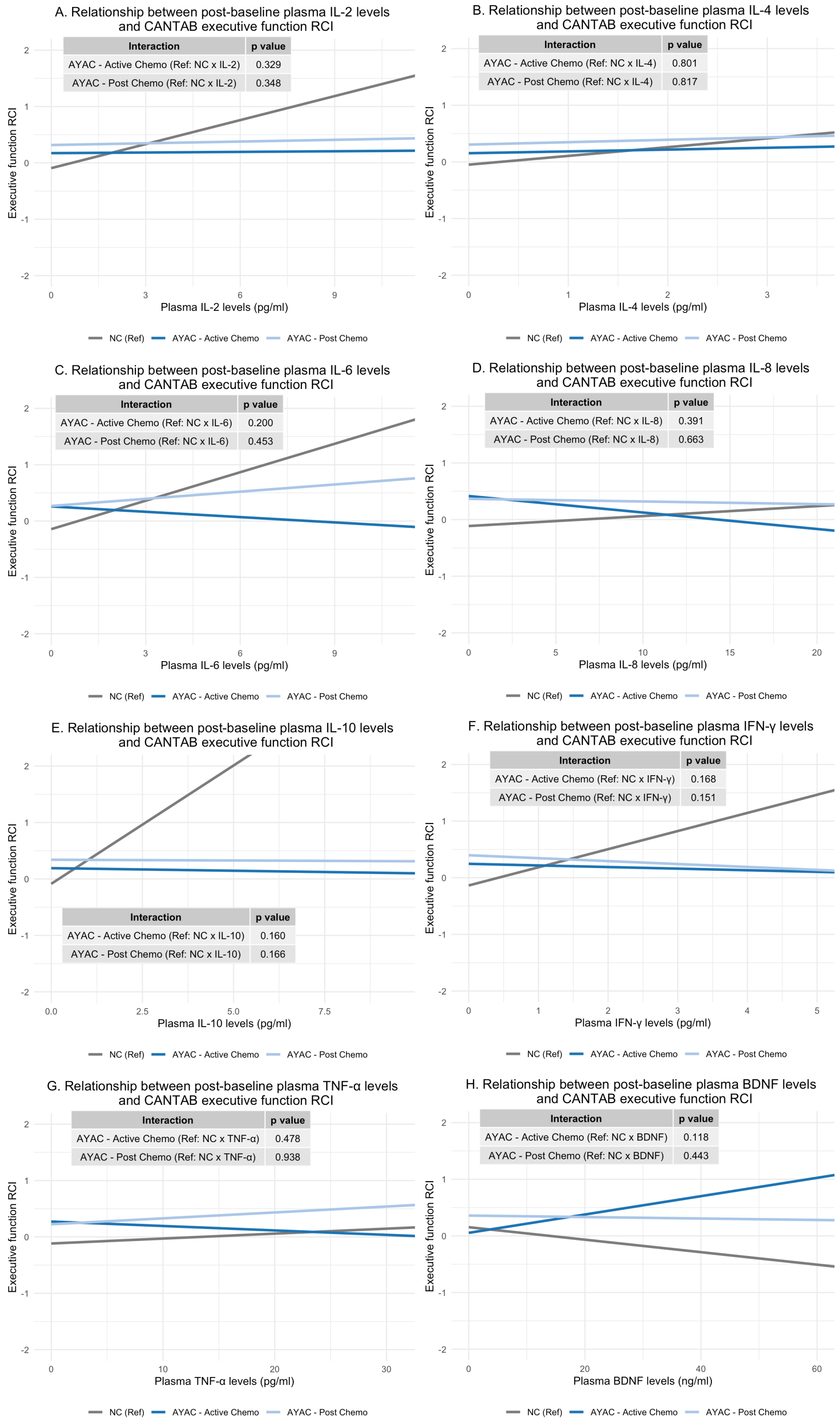

Supplementary Figure 6. Associations between biomarkers with executive function among NC and AYAC. Interaction plots from linear mixed models investigating the associations of (A) IL-2, (B) IL-4, (C) IL-6, (D) IL-8, (E) IL-10, (F) IFN-γ, (G) TNF-α, and (H) BDNF with CANTAB executive function RCI in AYAC and NC. The analysis was performed with interaction terms for each biomarker and group (AYAC stratified by chemotherapy status vs. NC) in predicting cognition with adjustments for baseline cognition, sociodemographic variables, fatigue, and psychological distress. A negative RCI indicates cognitive decline from baseline. *AYAC* = adolescent and young adult cancer patients; *NC* = non-cancer controls; *IL* = interleukin; *TNF-α* = tumor necrosis factor alpha; *IFN-γ* = interferon gamma; *BDNF* = brain-derived neurotrophic factor; *CANTAB* = Cambridge Neuropsychological Test Automated Battery; *RCI* = reliable change index.

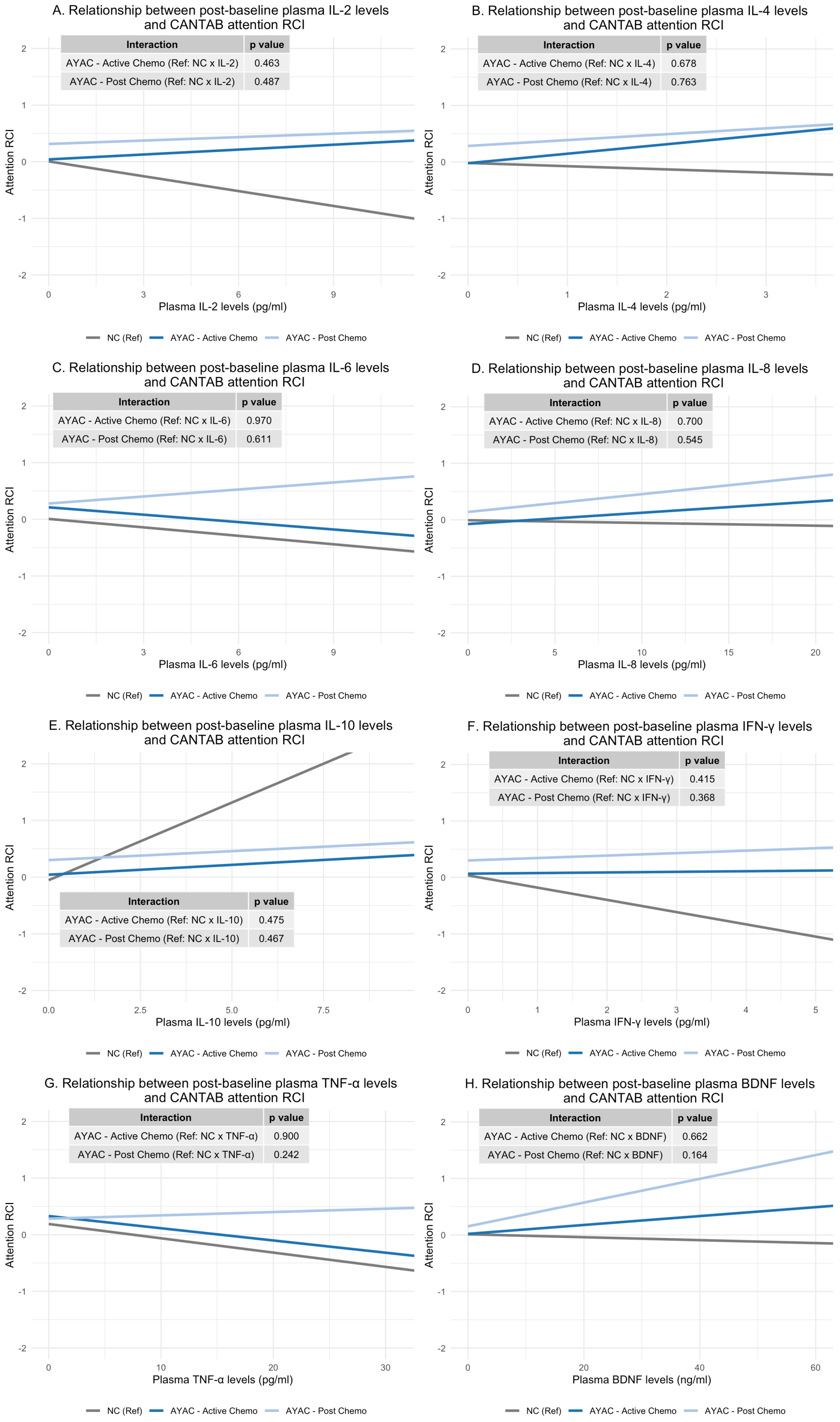

Supplementary Figure 7. Associations between biomarkers with attention among NC and AYAC. Interaction plots from linear mixed models investigating the associations of (A) IL-2, (B) IL-4, (C) IL-6, (D) IL-8, (E) IL-10, (F) IFN-γ, (G) TNF-α, and (H) BDNF with CANTAB attention RCI in AYAC and NC. The analysis was performed with interaction terms for each biomarker and group (AYAC stratified by chemotherapy status vs. NC) in predicting cognition with adjustments for baseline cognition, sociodemographic variables, fatigue, and psychological distress. A negative RCI indicates cognitive decline from baseline.

AYAC = adolescent and young adult cancer patients; *NC* = non-cancer controls; *IL* = interleukin; *TNF-α* = tumor necrosis factor alpha; *IFN-γ* = interferon gamma; *BDNF* = brain-derived neurotrophic factor; *CANTAB* = Cambridge Neuropsychological Test Automated Battery; *RCI* = reliable change index.

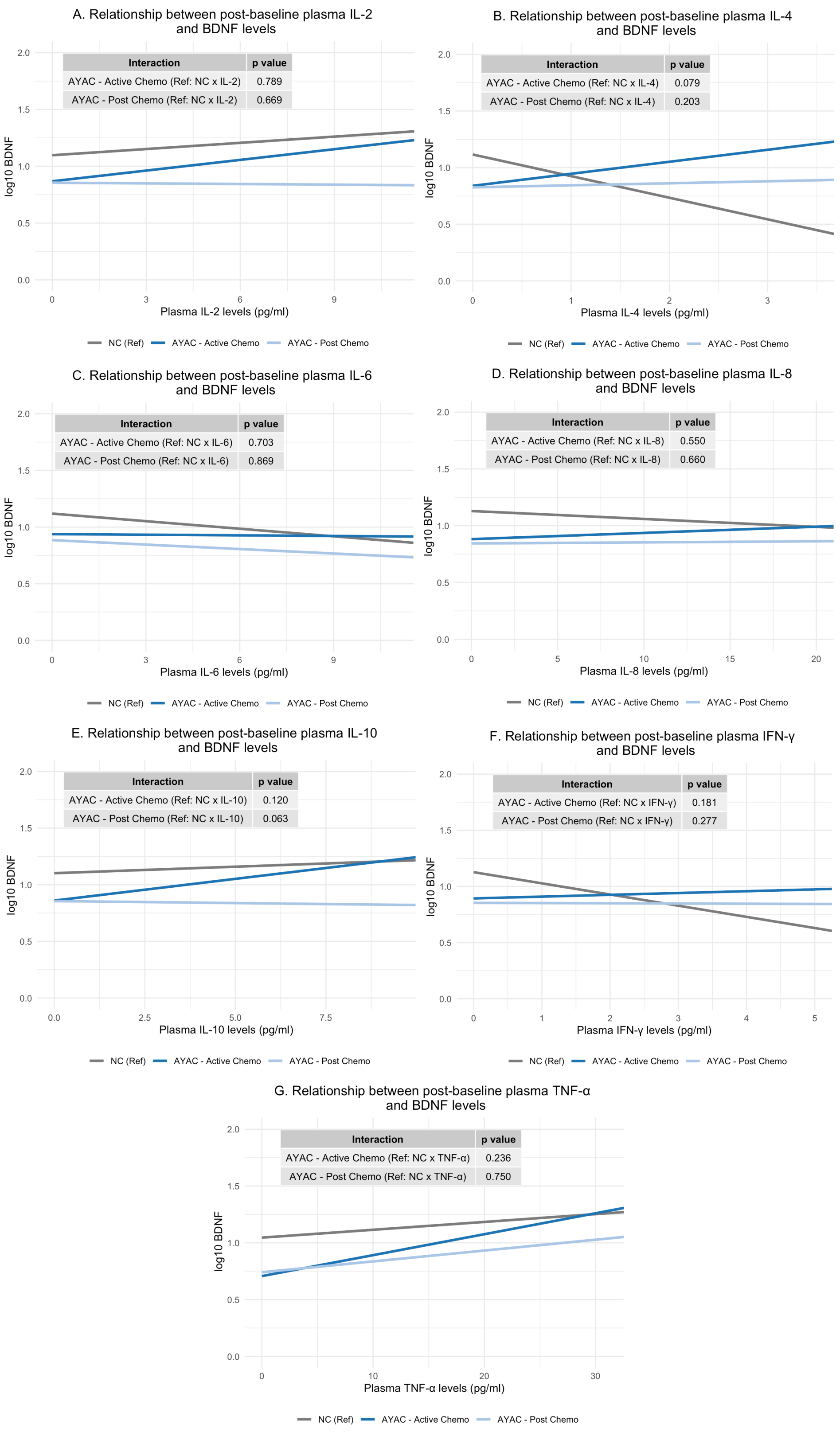

Supplementary Figure 8. Cytokine-BDNF associations among AYAC and NC. Interaction plots from linear mixed models investigating the associations of (A) IL-2, (B) IL-4, (C) IL-6, (D) IL-8, (E) IL-10, (F) IFN-γ, and (G) TNF-α with BDNF in AYAC and NC. The analysis was performed with interaction terms for each cytokine and group (AYAC stratified by chemotherapy status vs. NC) in predicting $\log_{10}$BDNF levels with adjustments for baseline BDNF, sociodemographic variables, and BDNF Val66Met (rs6265) genotype.
AYAC = adolescent and young adult cancer patients; *NC* = non-cancer controls; *IL* = interleukin; *TNF-α* = tumor necrosis factor alpha; *IFN-γ* = interferon gamma; *BDNF* = brain-derived neurotrophic factor
